# Factors associated with localization of tuberculosis disease among patients in a high burden country: A health facility-based comparative study in Ethiopia

**DOI:** 10.1016/j.jctube.2021.100231

**Published:** 2021-03-24

**Authors:** Hawult Taye, Kassahun Alemu, Adane Mihret, James L.N. Wood, Ziv Shkedy, Stefan Berg, Abraham Aseffa, the ETHICOBOTS consortium

**Affiliations:** a Armauer Hansen Research Institute, Addis Ababa, Ethiopia; b Department of Epidemiology and Biostatistics, University of Gondar, Gondar, Ethiopia; d Department of Veterinary Medicine, University of Cambridge, Cambridge, UK; c Biostatistics and Bioinformatics, University of Hasselt, Belgium; e Bacteriology Department, Animal and Plant Health Agency, Weybridge, UK

**Keywords:** Tuberculosis, Clinical disease category, Associated factors

## Abstract

**Introduction:**

In contrast to most tuberculosis (TB) high burden countries, Ethiopia has for a long time reported a very high percentage of extra pulmonary TB (EPTB), which is also reflected in population based estimations reported by the World Health Organization (WHO). Particularly a steadily higher proportion of cervical tuberculous lymphadenitis (TBLN) has been described. Here we identify clinical and demographic factors associated with anatomic site of the TB disease.

**Method:**

A health facility based comparative study was conducted among TBLN and PTB patients who visited selected health facilities in Ethiopia during 2016 and 2017. Associated risk factors were identified through a multivariate logistic regression model using R-studio.

**Result:**

A total of 1,890 study participants, 427 TBLN and 1,463 PTB patients, were included. The mean age of TBLN patients (29 years ± 14.4 SD) was lower than that of PTB cases (36 years ± 15.0 SD). There were slightly more women diagnosed with TBLN (51.1%) while nearly 6 out of 10 male patients were diagnosed with PTB (58.9%). Most significantly, younger age groups (<15 Years) were more likely to develop cervical TBLN than older people (>56 years), with an AOR of 9.76 (95% CI: 4.87, 19.56). The odds of cervical TBLN among women [1.69 (1.30, 2.20)] was higher than that for men. In addition, adjusted estimates suggested that, compared with PTB, renal diseases [3.41 (1.29, 9.02)] and the presence of other concomitant chronic illness [1.61 (1.23, 2.09)] had a significant association with TBLN.

**Conclusion:**

Generally, the risk of developing a particular form of TB disease is usually associated with demographic and medical history of an infected individual. Hence, the current symptom based screening, which primarily rely on chronic cough in many countries, may lead to missing significant portions of TBLN cases.

## Highlights

• A steady high rate of cervical tuberculous lymphadenitis (TBLN) has been reported in Ethiopia.
• That was significantly associated with biological factors that include age and sex of individuals.
• The risk of developing TBLN also related with chronic medical conditions such as renal disease.
• Suggests that specific symptom based screening may lead to missing a significant portion of cases.
• Hence, lymph node enlargement should be considered in the national case screening program.

## 1 Introduction

Global epidemiology of tuberculosis (TB) have shown huge prevalence difference across regions, and with the majority of TB cases being from South-East Asia (45%) and Africa (25%) [1]. About 87% of the total incidence cases were from only 30 high burden countries [2]. In reverse relationship with the crude prevalence, however, relatively higher percentage of EPTB cases were estimated from many low burden settings [3], [4], [5], suggesting that, in countries with successful TB control programs, the incidence of PTB but not EPTB cases have significantly decreased during such programs [3], [6]. In contrast to many high burden countries, high percentage of EPTB were reported in Ethiopia [5], [7]. It comprises up to 30–40% of the total TB cases registered annually in the country while the overall average global proportion of EPTB is about 15% [5]. In agreement with the above WHO estimate, the recent (2016) national case notification survey identified about 32.4% and 43.8% of EPTB cases in Oromia and Amhara, respectively, the two largest regions of Ethiopia [8]. It has been clearly and frequently noted that TBLN accounts for the majority of EPTB cases, with a steady high rate of TBLN [9], [10].

Because of high rate of transmissibility, TB control program has apparently focused on tracing PTB cases [11]. Nevertheless, TB disease transmission and progression of TB within an individual is a result of complex dynamics usually connected with behavioural, medical and/or biologic risk factors [12], [13], [14]. In some circumstances, the proportion of PTB among men could be more than two fold compared to women. This is often associated with smoking and comorbidities of chronic pulmonary disease [13], [15]. Concerning known chronic diseases, HIV and renal disease have been associated with an increased prevalence of EPTB [16], [17] while diabetes mellitus (DM) is mainly related to PTB [18], [19].

From a broader perspective, the rate of TB disease and its proportion of cases in a given community is a cumulative effect of the above mention dynamics [20]. Data driven prediction model by Iglesias et al. (2018) has shown that the demographic dynamicity (transition) and contact pattern heterogeneity within specific age groups will impact the overall TB burden in a given community [14]. Similarly, other interrelated biological or medical and most behavioural risk factors were assumed to be transient across population groups. As a consequence of that, there is always a shift in the epidemiology and clinical form of active TB disease [21]. However, the reason why an individual has experienced a particular type of active TB infection and forecasting the proportion of cases with respect to a specific community or region remains unclear. Hence, investigating the clinical and epidemiological aspects of TB is of continuing importance. This study aimed to identify demographic and clinical factors that could be associated with an increased prevalence of TBLN diseases in Ethiopia as compared to PTB.

## 2 Methodology

### 2.1 Study design and setting

A multicentre cross-sectional comparative study was conducted in Ethiopia during 2016 and 2017, as part of the Ethiopia Control of Bovine Tuberculosis Strategies (ETHICOBOTS) project, designed to explore control strategies for bovine TB in Ethiopia. Four hospitals, two private clinics, and fourteen health centres located in urban and *peri*-urban areas, were purposively selected from four different regions with either an established or an emerging dairy sector. Addis Ababa and the surrounding special zone of Oromia region constituted the largest study site (a well-established dairy belt) while the remaining three study sites (urban cities) of ETHICOBOTS were Gondar in Amhara, Hawassa in Southern Nations Nationalities, and Peoples’ region, and Mekele in Tigray region, all with emerging dairy sectors.

### 2.2 Study population

Although one of the main aims of the ETHICOBOTS study was intended to estimate the prevalence of zoonotic TB (rate of *M. bovis* infection in human), recruitment of participants from selected health facilities were done according to the routine clinical case definition criteria. All clinically diagnosed and presumed TB cases were initially considered as potential sources of study population. In the routine healthcare practices, cases were classified into different categories according to the national guideline standard case definition criteria [7]. Both PTB and TBLN cases, who were eligible for first-line Anti-TB treatment, were asked for consent and consecutively enrolled. EPTB other than TBLN and those who were clinically diagnosed as PTB but also presented with cervical node swelling were excluded. For that reason, the abbreviated term “TBLN” used in this study, represent cervical tubercular lymphadenitis alone. Patients who had already started anti-TB treatment and any known multi-drug resistant (MDR) cases were not included.

### 2.3 Case classification and study variables

Patients who had disease apparent within the lung parenchyma alone were classified as “PTB,” while cases with TB exclusively involving cervical lymph nodes were classified as “cervical TBLN”. For the first group, case classification was done by clinicians supported by bacteriologic investigation (Ziehl-Neelsen staining) and/or radiologic examination. Because of limited diagnostic facilities and skilled professionals, FNAC examination was not performed at governmental health centers. Hence, the second group (TBLN) of patients were recruited from pathology units of selected hospitals and private clinics. Like that of PTB, case classification was done by physicians and supported by fine needle aspirate cytology (FNAC) and smear microscopy (Ziehl-Neelsen staining) results.

Clinical and demographic variables were collected using a predesigned structured questionnaire. Socio-demographic risk factors (age, sex, address, religion, educational status, occupation, income, family size) were recorded through the patient interview. Similarly, although, most of the information has been documented as part of the routine clinical evaluation procedure, questionnaire based patient interview was done to collect current and past medical history of the study participants. These included history of TB, BCG vaccination status, HIV status, known non-communicable chronic diseases (NCD) and the presence of other chronic concomitant infection (disease).

### 2.4 Data management and statistical analysis

All data were double entered in to a pre-design database (Open Clinica) and the statistical analyses were performed using SPSS and the R-package (Studio). Logistic regression was applied to identify factors associated with site of active TB disease development. Multivariate analysis was executed to control for overlapping (confounding) effect of clinical and demographic variables. Initially, all clinically relevant factor variables were included in the full model. Then using the specific statistical command (Step) under R-studio, the software program automatically generated all possible alternative models having lists of dependent and independent variables. Using this stepwise backward elimination technique and according to Likelihood Ratio-test and model with potential explanatory variables that has the lowest AIC (Akaki information criteria) were selected. Finally, only adjusted odds ratio (AOR) with 95% CI and respective P-value of each predictor variables were reported.

## 3 Results

### 3.1 Demographic characteristics of patients

**Fig. 1:**
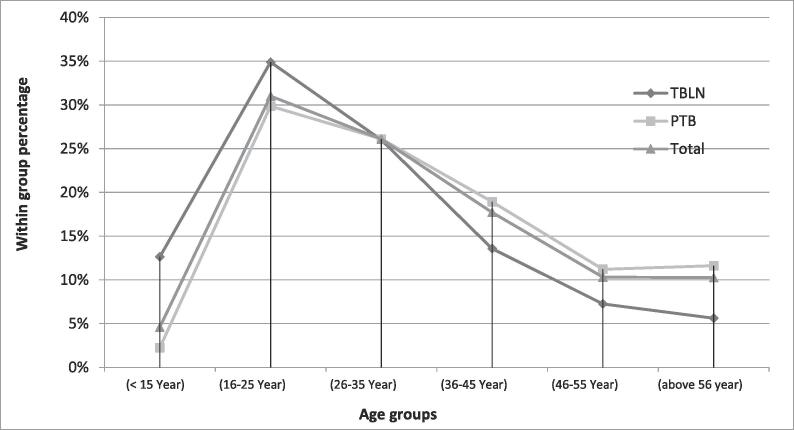
Line chart shows relative proportions (within age group percentage) of cervical tuberculous lymphadenitis (TBLN) and pulmonary tuberculosis (PTB) patients recruited at selected health facilities in Ethiopia in the years of 2016/17.

A total of 1,890 study participants, 427 TBLN and 1,463 PTB patients, were included. The mean age of TBLN patients (29 years ± 14.4 SD) was lower than that of PTB cases (36 years ± 15.0 SD). Correspondingly, the mean BMI of TBLN cases (16.6 ± 1.0) was lower than for PTB (18.2 ± 2.6). There were slightly more women diagnosed with TBLN (51.1%), while nearly 6 out of 10 patients diagnosed with PTB (58.9%) were male (Table 1). As presented in Fig. 1, the line chart shows a relatively higher percentage of TBLN cases among younger age groups (age ≤ 35 Year) and then the trend decreased in the later age groups, older people (age > 35 years).

**Table 1:** Sociodemographic characteristics of cervical tuberculous lymphadenitis (TBLN) and pulmonary tuberculosis (PTB) patients recruited at selected health facilities in Ethiopia in the years of 2016/17.

Characteristics of participants	TBLN = (4 2 7) No (%)	PTB = (1463)No (%)	Total = (1890) No (%)	P-value of X^2^ –test
Age group				< 0.001
(<15 Year)	54 (12.6)	33 (2.3)	87 (4.6)	
(16–25 Year)	149 (34.9)	437 (29.9)	586 (31)	
(26–35 Year)	111 (26)	382 (26.1)	493 (26.1)	
(36–45 Year)	58 (13.6)	277 (18.9)	335 (17.7)	
(46–55 Year)	31 (7.3)	164 (11.2)	195 (10.3)	
(>56 year)	24 (5.6)	170 (11.6)	194 (10.3)	
Gender				< 0.001
Male	209 (48.9)	862 (58.9)	1071 (56.7)	
Female	218 (51.1)	601 (41.1)	819 (43.3)	
J Journal of Steroids				0.558
Illiterate	185 (43.3)	643 (44.0)	828 (43.8)	
Primary	131 (30.7)	488 (33.4)	619 (32.8)	
Secondary	74 (17.3)	227 (15.5)	301 (15.9)	
Diploma	19 (4.4)	60 (4.1)	79 (4.2)	
Degree & above	18 (4.2)	44 (3)	62 (3.3)	
Religion				0.004
Orthodox	286 (23.2)	949 (76.8)	1235 (65.3)	
Muslim	56 (30.8)	126 (69.2)	182 (9.6)	
Protestant	81 (18.2)	364 (81.8)	445 (23.5)	
Catholic	3 (11.5)	23 (88.5)	26 (1.4)	
Others	1 (50.0)	1 (50.0)	2 (0.1)	
Marital status				< 0.001
Single	200 (46.8)	477 (32.6)	677 (35.8)	
Married	200 (46.8)	844 (57.7)	1044 (55.2)	
Separated	6 (1.4)	24 (1.6)	30 (1.6)	
Divorced	11 (2.6)	50 (3.4)	61 (3.2)	
Widowed	10 (2.3)	68 (4.6)	78 (4.1)	
Occupation				< 0.001
Farmer	142 (33.5)	485 (33.3)	627 (33.3)	
Merchant	22 (5.2)	133 (9.1)	155 (8.2)	
Employee	68 (16.0)	180 (12.3)	248 (13.2)	
Student	81 (19.1)	194 (13.3)	275 (14.6)	
House wifve	65 (15.3)	277 (19.0)	342 (18.2)	
Dairy worker	11 (2.6)	89 (6.0)	99 (5.3)	
Others	35 (8.3)	101 (6.9)	136 (7.2)

### 3.2 Medical history of study participants

**Fig. 2:**
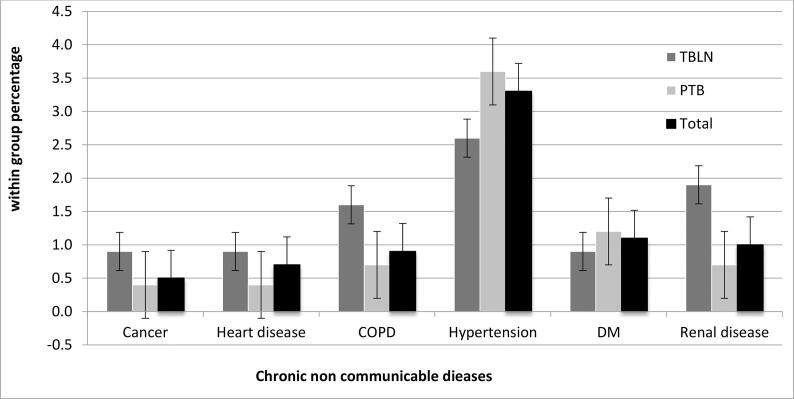
Bar graph comparing the proportion (within group percentage) of chronic non communicable diseases (NCDs) among cervical tuberculosis lymphadenitis (TBLN) and pulmonary tuberculosis (PTB) patients recruited at selected health facilities in Ethiopia in the years of 2016/17.

Comparing the two groups, 231/1,463 (14.6%) of PTB and 108/427 (25.3%) of TBLN patients reported that they had been vaccinated with BCG. Out of all participants, about 13.7% had history of TB with a relatively higher percentage (16%) of PTB cases recalled past diagnosis and Anti-TB treatment history while only 5.9% of TBLN cases did so. At the time of their diagnosis, 342/1,463 (23.4%) of PTB and 138/427 (32.3%) of TBLN cases recognized the presence of at least one additional concomitant chronic disease. Although, there was a very low self-reported history of known NCDs (Fig. 2), the prevalence of DM and hypertension was higher among PTB patients, while the proportion of renal disease was predominantly higher among TBLN cases.

### 3.3 Factors associated with localization of TB disease

According to the multivariate logistic regression model, sociodemographic factors such as age, gender, occupation, BMI and income were independently associated with the type of TB disease presented (Table 2). Most significant, age and gender showed strong connection to anatomic site of active TB disease. For instance, compared to the oldest age group (>56 years), the odds of developing TBLN were significantly higher among youngest age group (<15 years) with an AOR of 9.76 (4.87, 19.56)]. Regarding gender, females were at higher risk of TBLN than male [1.69 (1.30, 2.20)]. With respect to participants occupation, compared to employed workers, the odds of TBLN was significantly lower among merchants [0.45 (0.26, 0.79)], students [0.63 (0.41, 0.98)], house wives [0.55 (0.35, 0.87)] and dairy worker [0.33 (0.16, 0.68)]. Adjusted odds ratios reflected that among patients with known NCDs, only those with renal diseases showed a significant difference. Here TBLN patients were more likely to have an associated chronic renal diseases than PTB cases [AOR: 3.41 (1.29, 9.02)]. Similarly, the risk of developing TBLN had significantly increased among patients having additional concomitant chronic illness (other than known NCDs) [AOR: 1.61 (1.23, 2.09)] (Table 2).

**Table 2:** Multivariate logistic regression analysis used to identify risk factors contribute for cervical tuberculous lymphadenitis (TBLN) relative to pulmonary tuberculosis (PTB).

Study Variables	TBLN	PTB	COR (95% CI)	P-value	AOR (95% CI)	P-value
Age group						
(<15 Year)	54(62.1)	33(37.9)	11.59 (6.31,21.3)	< 0.001	9.76 (4.87,19.56)	< 0.001
(16–25 Year)	149(25.4)	437(74.6)	2.42 (1.52,3.85)	< 0.001	2.33 (1.40,3.90)	0.001
(26–35 Year)	111(22.5)	382(77.5)	2.06 (1.28,3.32)	0.003	2.21 (1.34,3.65)	0.002
(36–45 Year)	58(17.3)	277(82.7)	1.48 (0.89,2.48)	0.132	1.65 (0.97,2.80)	0.066
(46–55 Year)	31(15.9)	164(84.1)	1.34 (0.75,2.38)	0.319	1.47 (0.81,2.65)	0.207
(>56 year)	24(12.4)	170(87.6)	Ref		Ref	
Gender						
Male	209(19.5)	862(80.5)	Ref		Ref	
Female	218(26.6)	601(73.4)	1.50 (1.20,1.86)	< 0.001	1.69 (1.30,2.20)	< 0.001
Occupation						
Farmer	142 (22.6)	486 (77.4)	0.77 (0.55,1.07)	0.119	1.03 (0.71,1.50)	0.862
Merchant	22 (14.2)	133 (85.8)	0.43 (0.26,0.74)	0.002	0.45 (0.26,0.79)	0.005
Employee	69 (27.6)	181 (72.4)	Ref		Ref	
Student	83 (30.0)	194 (70.0)	1.12 (0.77,1.64)	0.550	0.63 (0.41,0.98)	0.038
House wife	65 (18.9)	279 (81.1)	0.61 (0.42,0.90)	0.013	0.55 (0.35,0.87)	0.010
Dairy worker	11(11.0)	89 (89.0)	0.32 (0.16,0.64)	0.001	0.33 (0.16,0.68)	0.003
Others	35 (25.7)	101 (74.3)	0.91 (0.57,1.46)	0.693	0.73 (0.43,1.22)	0.230
Religion						
Orthodox	286 (23.2)	949 (76.8)	Ref		Ref	
Muslim	56 (30.8)	126 (69.2)	1.47 (1.05,2.07)	0.026	1.84 (1.25,2.71)	0.002
Protestant	81 (18.2)	364 (81.8)	0.74 (0.56,0.97)	0.030	0.69 (0.50,0.94)	0.017
Catholic	3 (11.5)	23 (88.5)	0.43 (0.13,1.45)	0.175	0.42 (0.12,1.45)	0.171
BCG vaccination history						
No	181 (23.8)	579 (76.2)	Ref		Ref	
Yes	108 (33.6)	213 (66.4)	1.62 (1.22,2.16)	< 0.001	1.37 (0.98,1.91)	0.063
I don’t Know	138 (17.1)	671 (82.9)	0.66 (0.51,0.84)	< 0.001	0.75 (0.57,0.99)	0.039
History of past TB treatment						
No	376 (24.1)	1182(75.9)	Ref		Ref	
Yes	25 (9.7)	234 (90.3)	0.34 (0.22,0.52)	< 0.001	0.36 (0.23,0.56)	< 0.001
I don’t Know	26 (35.6)	47 (64.4)	1.74 (1.06,2.85)	0.028	2.42 (1.43,4.12)	0.001
Chronic Renal diseases						
No	419 (22.4)	1453(77.6)	Ref		Ref	
Yes	8 (44.4)	10 (55.6)	2.77 (1.09,7.07)	0.033	3.41 (1.29,9.02)	0.013
Concomitant chronic illness						
No	289 (20.5)	1121(79.5)	Ref		Ref	
Yes	138 (28.7)	342 (71.2)	1.57 (1.24,1.98)	< 0.001	1.61 (1.23,2.09)	< 0.001

## 4 Discussion

This study set out to determine important demographic and clinical factors associated with the most common type of TB disease. In agreement with other research findings, this study suggests that compared to PTB the odds of developing TBLN decrease as age increases [6], [10], [13], [22]. One of the practical reasons for the above difference could be the possibility of more missed childhood cases of PTB than of cervical TBLN. Inability to produce sputum samples and the presence of non-specific clinical symptoms of PTB has been masking the diagnosis of PTB in children [23]. On the other hand, in contrast to findings and reports from high TB burden countries, higher percentage of older patients with EPTB were observed in low burden settings, for example Spain [24] and USA [23]. This might be due to a difference in age structure of the population and the lower risk of active infection transmission in low burden countries [4].

In favour of our finding, there are many published reports supporting that females were more likely to develop TBLN while men were at higher risk of getting PTB [13], [15], [22]. Nevertheless, as for age, the reason for the greater risk of TBLN among females is not clearly understood, but the effect of cellular immunity, hormones and other confounding social and behavioural factors have been linked to these differences [15]. Previous studies have demonstrated that a higher prevalence of smoking and related concomitant pulmonary diseases among men could increase the risk of develop PTB more easily than women [24], [25].

In high burden settings, greater risk of PTB were perceived from congregated atmospheres where contact with an index case is prominent [26]. In this connection, our multivariate analysis showed that, compared to employed workers, the odds of TBLN was significantly lower among merchants, students and housewives. On the contrary, Memish et al. (2014) found greater proportions of EPTB cases among housewives and workers sharing the same household environment than those who were self-employed [6]. These contrary findings might be the effect of confounding and the bivariate logistic regression model used by Memish et al. is understandably limited to control for possible effects of gender and age.

The average proportion (13%) related with history of TB was much higher than previous research findings. For instance, a ten years retrospective study by Dangisso et al. (2014) found the proportion of case retreatment to range from 3% up to 7% [27]. The average percentage of retreatment case estimated from a fifteen-year retrospective study in central Ethiopia was nearly 7% of the total number of TB cases [28]. However, higher rate of retreatment has recently been reported in Adama, Ethiopia with a significant increment within two years, from 13% in 2013 to 29% in 2015 [29]. During our data collection, we noted that some of the study participants were not able to remember whether they had previously been treated with full course of Anti-TB drugs or not. In addition, further descriptive analysis informed that the majority of cases had their first TB diagnose many years ago (average duration of five years). This is in agreement with Dangisso et al. (2018) who showed in an Ethiopian study that three-fourth of TB recurrence occurred during the first 5 years of the post-treatment period [30]. However, because of a notable recall bias on treatment in our study, the higher rate of recurrent TB among the study population might not be due to either treatment failure or relapse but reinfection could rather have a significant impact [31].

Comorbidity of chronic disease was another area that we wanted to examine among clinically diagnosed TB cases. However, because of the small sample size, our multivariate analysis could not detect the true effect of known NCDs. In this regard, only renal disease and the presence of other concomitant chronic illness were found to have significant association with cervical TBLN. Previous research has stated that not being diabetic and having the end-stage renal disease were independent risk factors for EPTB [15]. Qian et al. (2018) recognized the relationship between end-stage renal disease and EPTB while compared to exclusive PTB cohorts, and they found the risk of developing EPTB among DM patients was significantly lower than for PTB patients [32]. Likewise, out study also observed a higher percentage of DM in PTB cases (Fig. 2). So far there are more pieces of evidence suggesting coincidence TB and other chronic NCDs such as cancer [33], but their independent relationship with respect to site of TB infection were not well investigated.

Finally, several notable limitations in this study need to be considered. First, it was limited by the use of a cross-sectional design as it is open for a number of biases such as selection (case classification) and recall bias. Because of lack of diagnostic facilities, enrolment of TBLN cases was restricted to three governmental hospitals and two private clinics, whereas recruitment of PTB cases was done at all study sites. That affected the proportion of cases between the two groups, and led to dispersed data distribution within some categorical factor variables. As a result, the observed insufficient measures reduced the analytical power. We did not confirm the status of known chronic comorbidities because of lack of standard clinical diagnostic methods or screening tests. On the other hand, many of the participants were not aware of their general medical status. Hence, failure to perform confirmatory tests could have led to profound recall bias or misclassification of cases.

However, we enrolled large numbers of participants from a diversified population, and considered the effect of all possible covariant variables during analysis; using stratification and multivariate modelling techniques. All inferential statistical estimates were adjusted values generated from a model with better explanatory power. Hence, we believe that some of the notified biases and confounding effects were minimized during analysis. Therefore, the estimates generated from our multivariate logistic regression model can reflect the overall situation in the target population and similar settings.

## 5 Conclusion

Although there are a number of preconditions that contributed to the overall diseases burden for a given community, localization and development of a particular form of TB disease is often directly connected with individual level risk factors. Thus, the risk for developing a particular form of TB disease is usually associated with demographic and medical history of an infected individual. The relevance of age, sex and chronic medical conditions such as renal disease and other concomitant illness in relation to the form of TB was shown in the present study. This suggests thatspecific symptom based screening which primarily relies on chronic cough may lead to missing a significant proportion of TBLN cases. Rather, it is better to give more attention for individual-centred healthcare programs that should target all potential high risk groups flexibly depending on individual level risk factors. Particularly, related clinical characteristics of lymph node enlargement should be considered as one of the symptom based screening criteria in the national case identification program. However, to figure out the most influential biologic and clinical factors for progression of specific form of TB, there is a need for further clinical and epidemiological investigations.

## CRediT authorship contribution statement

**Hawult Taye:** Conceptualization, Project administration, Data curation, Methodology, Formal analysis, Writing - original draft, Writing - review & editing. **Kassahun Alemu:** Supervision, Methodology, Writing - review & editing. **Adane Mihret:** Project administration, Data curation, Writing - review & editing. **James L.N. Wood:** Funding acquisition, Project administration, Writing - review & editing. **Ziv Shkedy:** Supervision, Methodology, Writing - review & editing. **Stefan Berg:** Funding acquisition, Conceptualization, Project administration, Data curation, Writing - review & editing. **Abraham Aseffa:** Funding acquisition, Conceptualization, Project administration, Methodology, Writing - review & editing. **:** .

## Declaration of Competing Interest

The authors declare that they have no known competing financial interests or personal relationships that could have appeared to influence the work reported in this paper.
